# Scirrhous Cancer in a Cow

**Published:** 1881-01

**Authors:** 


### IV.—SCIRRHOUS CARCINOMA IN A COW.

At a recent meeting of the Pathological Society of New York, Dr. Satterthwaite presented a specimen of scirrhous cancer from a cow. The history, furnished by J. Lindsay, D. V. S., of Huntington, Long Island, was as follows:

In January last a cow, six years old, and in excellent condition, received a blow on the side of the cheek. In April following a tumor was first detected at the spot where the injury had been received. It grew; the adjacent lymphatic glands became involved, and also the eye. In June there was a discharge from the growth and its extension became more rapid. Finally, as there was no amelioration in the symptoms, the animal was killed by advice of Dr. Lindsay, who diagnosticated carcinoma, and gave an unfavorable prognosis.

The tumor, when removed, was found to measure fourteen inches in its greatest circumference and five inches in diameter, with a maximum thickness of two inches. It was surrounded by a slight fibrous capsule, something of a rarity in these malignant neoplasms. On dissection, it exhibited the peculiarly white and glistening cords that belong to scirrhous, and it had the same creaking feel when cut into by the knife. Altogether the microscopic appearances were such as are commonly seen in scirrhous carcinoma of the female (human) breast.

The microscopic evidences of this particular variety of carcinoma were most satisfactory.

